# Regulation of Intestinal Glucose Absorption by Ion Channels and Transporters

**DOI:** 10.3390/nu8010043

**Published:** 2016-01-14

**Authors:** Lihong Chen, Biguang Tuo, Hui Dong

**Affiliations:** 1 Department of Gastroenterology, Affiliated Hospital, Zunyi Medical College, and Digestive Disease Institute of Guizhou Province, Zunyi 563003, China; 18786841557@163.com; 2 Department of Gastroenterology, Xinqiao Hospital, Third Military Medical University, Chongqing 400037, China

**Keywords:** glucose uptake, Na^+^/glucose cotransporter, Ca^2+^ channels, K^+^ channels, Na^+^/Ca^2+^ exchanger, Na^+^/H^+^ exchanger, glucose transporter type 2

## Abstract

The absorption of glucose is electrogenic in the small intestinal epithelium. The major route for the transport of dietary glucose from intestinal lumen into enterocytes is the Na^+^/glucose cotransporter (SGLT1), although glucose transporter type 2 (GLUT2) may also play a role. The membrane potential of small intestinal epithelial cells (IEC) is important to regulate the activity of SGLT1. The maintenance of membrane potential mainly depends on the activities of cation channels and transporters. While the importance of SGLT1 in glucose absorption has been systemically studied in detail, little is currently known about the regulation of SGLT1 activity by cation channels and transporters. A growing line of evidence suggests that cytosolic calcium ([Ca^2+^]_cyt_) can regulate the absorption of glucose by adjusting GLUT2 and SGLT1. Moreover, the absorption of glucose and homeostasis of Ca^2+^ in IEC are regulated by cation channels and transporters, such as Ca^2+^ channels, K^+^ channels, Na^+^/Ca^2+^ exchangers, and Na^+^/H^+^ exchangers. In this review, we consider the involvement of these cation channels and transporters in the regulation of glucose uptake in the small intestine. Modulation of them may be a potential strategy for the management of obesity and diabetes.

## 1. Introduction

Obesity or overweight is associated with a high risk of many diseases, such as ischemic heart disease, diabetes mellitus, hypertension, hyperlipidemia and cancers. Obesity and its related metabolic disorders are a global pandemic and the obese group has shorter life expectancy than normal of about 10 to 20 years. In the United States, around 71% of the population is either overweight or obese [1]. Therefore, healthy weight loss is a serious worldwide problem that urgently needs to be solved. At present, the major ways to prevent/treat obesity are the medications that mainly suppress appetite [2] (such as fluorine, benzedrine and sibutramine) or surgical procedures (such as gastric bypass surgery and gastrointestinal electrical stimulation, *etc.*). However, these ways will also disturb the absorption of other important nutrients, including vitamins, folic acid and so on, and finally may influence the balance of nutrients in the human body. Therefore, with a quick increase in the populations of overweight and obesity worldwide, it is urgent to investigate the detailed regulatory mechanisms of special nutrient absorption in order to find better strategies for fighting obesity.

Although obesity is a metabolic disease induced by multiple factors, it mainly results from an imbalance between energy intake and expenditure. Energy intake depends on the amount of nutrients absorbed by the brush border membrane of small intestinal epithelial cells (IEC) and on the amount of nutrients transported by mesenteric capillaries and lacteals. It has been generally accepted that excessive intake of glucose is one of the major sources for accumulating fat, and, therefore, selective inhibition of excessive glucose intake may disturb the absorption of other important nutrients less. Robert K. Crane originally described the cellular model of absorption of Na^+^ and glucose by a “co-transport process” in 1960 [3]. When the hypothesis of intestinal Na^+^/glucose co-transporter-mediated glucose absorption was proposed, the mechanisms of glucose absorption became a hot field of research. Afterwards, the SGLT1 gene was cloned on IEC and then most scholars focused their research on this transporter. Valentin Gorboulev and coworkers found that SGLT1^−/−^ mice lost their body weight [4], confirming its important role in the control of glucose absorption and, thus, body weight.

SGLT1 activity could be regulated by various factors, such as protein kinases [5]. Alexander used Ussing chamber technology to show that Insulin-like Growth Factors (IGF) affect jejunal glucose transport by PI3-kinase to stimulate Na^+^-K^+^-ATP-ase activity in enterocytes and enhanced Na^+^-couple glucose absorption [6]. Nevertheless, Casselbrant proposed that angiotension II combined with the angiotensin type 1 receptor (AT1R) or angiotensin type 2 receptor (AT2R), exerting the opposite action in mediating jejunal glucose/Na^+^ absorption [7]. Since the absorption of glucose is electrogenic [8] and mainly depends on the Na^+^ gradient across the epithelium, the changes in membrane potential of IEC can influence glucose absorption. Membrane potential is mainly controlled by activity of voltage-gated K^+^ channels (Kv). Decreasing the activity of Kv channels by applying Kv channel inhibitors results in membrane depolarization [9,10]. It is obvious that membrane depolarization or hyperpolarization can regulate the activity of SGLT1 on IEC. In addition to this aspect, as an important intracellular secondary messenger, Ca^2+^ regulates many physiological activities in the living cells, and plays a vital role in modulating intestinal absorption of glucose [11,12]. However, [Ca^2+^]_cyt_ is finely controlled by calcium channels and transporters [13,14], which may finally regulate glucose absorption. Therefore, it is the major aim of this review to summarize the current knowledge about intestinal glucose absorption and the regulation by epithelial cation channels and transporters.

## 2. Mechanisms of Glucose Absorption in the Intestine

**Figure 1:**
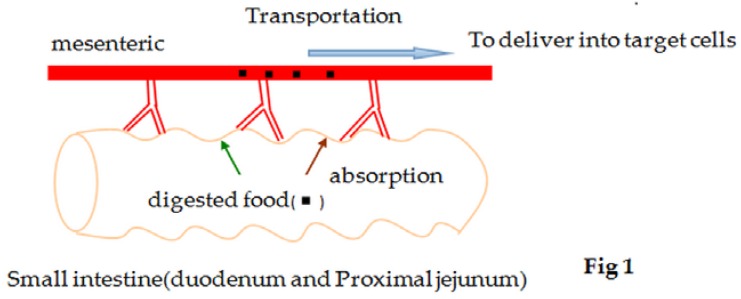
Absorption of digested food (glucose) from the intestinal lumen into the blood, and transportation of the absorbed nutrients via mesenteric circulation to target cells.

Complex carbohydrates reaching the small intestine must be hydrolyzed to monosaccharides such as glucose or galactose in order to be transported across the intestinal mucosa. The classical pathway of glucose absorption is across the intestinal brush-border membrane (BBM), which was predominantly mediated by SGLT1, a membrane protein that couples two molecules of Na^+^ together with one molecule of glucose. The passive move out of the basolateral surface of enterocytes contains a facilitated-diffusion glucose transporter (GLUT2) which allows glucose to move from the IEC into the extracellular medium near the blood capillaries [15] (Figure 1). The absorption of glucose may be adjusted by other transporters, such as GLUT2 [16,17,18]. Translocation of GLUT2 from cytoplasmic vesicles into the apical membrane markedly increases the capacity of glucose uptake by the enterocyte [19,20,21]. Thus, any factor that influences the activities of SGLT1 and GLUT2 will also alter the absorption and metabolism of glucose.

## 3. Detection Methods of Glucose Absorption *in Vitro*

At present, glucose absorption can be studied by two methods *in vitro* and *in vivo*. *In vitro* methods include tissue and cell experiments: (1) The Ussing chamber experiment uses intestinal tissues. Schultz and Zalusky were the first to use the short-circuit current to examine the electrical properties of rabbit ileum [22,23]. Specifically, they demonstrated via electrophysiological and radioisotopic experiments that the addition of glucose to the mucosal solutions resulted in a rapid increase in the transmural potential difference [24]. So this glucose-induced change in short-circuit currents was regarded as rates of glucose and Na^+^ transports across the epithelium; (2) Then there is the application of the isotope tracer method in intestinal tissue and IEC. d-(6-3H) Glucose or ^14^C glucose is used as the tracer to detect glucose absorption into intestinal tissue or IEC [25]; (3) To examine glucose absorption into a cell, glucose is absorbed into a cultural cell after glucose is added to the cell culture medium for a period of time, and the medium is then taken out for determining the concentrations of glucose by the hexokinase method or the glucose oxidizes/peroxides (GOD-POD) method [26].

## 4. Regulation of Glucose Absorption by Potassium Channels

### 4.1. Potassium Channels in Small Intestinal Epithelial Cells

The concentration of K^+^ inside the cell is roughly 20-fold larger than the outside. K^+^ channels function to conduct potassium ions down their electrochemical gradient to maintain ion equilibrium, and provide electrochemical driving force to maintain cell function [27,28,29]. K^+^ channels represent the largest and most heterogeneous family of ion channels and membrane proteins. They are widely expressed in both excitable and non-excitable cells [27,30,31]. In epithelial cells, K^+^ channels are expressed in a polarized fashion and serve two principal functions for transepithelial transports: the generation of membrane potential and the recycling of K^+^ [32]. As in duodenal epithelial cells, an intermediate-conductance Ca^2+^-activated K^+^ channel (IK_Ca_) can provide a driving force for duodenal bicarbonate secretion [33]. On the intestinal mucosa surface, intermediate conductance K^+^ channels (KCNN4) can provide a driving force for Cl^−^ secretion via both cystic fibrosis transmembrane conductance regulator(CFTR ) and Ca^2+^-activated Cl^−^ channels (CaCC) that are mediated by cAMP and Ca^2+^ [34]. K^+^ channels also regulate cell volume in isosmotic conditions in small intestinal epithelial cells [35]. Therefore, K^+^ channels may be involved in various physiological processes of small intestinal epithelial cells. Especially, we deal with the regulatory mechanism of glucose absorption by K^+^ channels.

### 4.2. Regulatory Mechanisms of Glucose Absorption by Potassium Channels

In the small intestine epithelial cells, K^+^ channels provide the driving force required for Na^+^-dependent uptake of glucose into IEC. The glucose uptake is driven by the Na^+^ transmembrane gradient and membrane potential (Em). Nevertheless, Em is primarily determined by plasmalemmal K^+^ channels; the inhibition of K^+^ channels (especially the voltage-gated potassium channels) in IEC would induce cell membrane depolarization and inhibit the nutrient absorption by reducing the driving force for Na^+^ [36]. It was found that chromanol 293B, a selective blocker of KCNQ1 expressed in IEC, can enhance glucose tolerance and glucose-stimulated insulin secretion and plasma GLP-1 levels in cultured islets and intact animals [37]. Activation of ATP-sensitive K^+^ channels (KATP) induces glucose-stimulated gastric inhibitory polypeptide release to finally affect glucose metabolism [38].

Hiroyuki Unoki identified KCNQ1 to be associated with susceptibility to type 2 diabetes [39,40]. Kv1.3^−/−^ mice gained significantly less weight than controls on a high-fat diet, but Kv1.3^+^/^+^ mice developed hyperglycemia [41]. There are different opinions as to how K^+^ channels play a role in regulating blood glucose concentration. The blockade of K^+^ channels in peripheral tissues is predominantly due to increasing peripheral insulin sensitivity, augmenting insulin release from pancreatic islets and increasing in basal metabolic rate [42,43,44]. However, some studies do not support a role for Kv1.3 channels in the regulation of glucose homeostasis or peripheral insulin sensitivity in mice or humans [45,46], but support their role in the regulation of obesity and diabetes by modulating electrophysiological characteristics of IEC.

**Figure 2:**
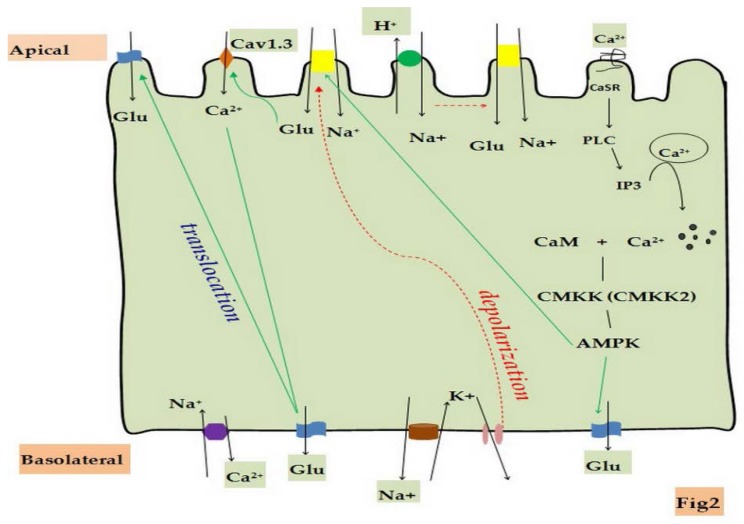
Schematic illustration of regulatory mechanisms of small intestinal glucose uptake by cation channels and transporters. Glucose absorption takes place in small intestinal villus cells by SGLT1, which is driven by active sodium extrusion via the basolateral Na^+^/K^+^ ATPase. When basolateral K^+^ channels are closed to depolarize the membrane voltage, the electrogenic transportation of glucose is blocked. Intestinal glucose absorption is also up-regulated by Ca^2+^-dependent apical GLUT2 insertion. The [Ca^2+^]_cyt_ is mainly excluded by NCX1 on the basolateral IEC. NHE3 suppresses SGLT1 and ultimately affects intestinal glucose absorption. Increased [Ca^2+^]_cyt_ through CaSR is combined with CaM as a complex, which modulates AMPK and further activates SGLT1 or GLUT2 to adjust small intestinal glucose absorption.

The electrophysiological studies indicate that KCNE1-dependent K^+^ movement from the cell to the lumen contributes to the maintenance of the electrical driving force for Na^+^-coupled transport in the proximal tubule [47]. However, K^+^ channels that regulate glucose absorption in the small intestine have long been overlooked. The presented model of the absorption of glucose provides insight into how intestinal epithelial cells maintain intracellular ion homeostasis (Figure 2). The driving force for this transport protein is the Na^+^ electrochemical gradient that is generated by the basolateral Na^+^/K^+^ ATPase [48]. Na^+^/K^+^ ATPase is an integral transmembrane protein in the basolateral membrane of mammalian IEC which extrudes three Na^+^ and takes in two K^+^. This mechanism aids in maintaining the negative membrane potential of IEC to facilitate Na^+^ entry across the luminal membrane, which drives the apical absorption of glucose in SGLT1 [49,50]. K^+^ electrochemical gradient also plays an important role in maintaining the activity of Na^+^/K^+^ ATPase and membrane potential. Intracellular K^+^ is recycled back across the basolateral membrane via K^+^ channels [51,52]. Karin Dedek demonstrated that KCNQ1 and KCNE3 are expressed in the basolateral localization of crypt cells of IEC and serve as a recycling pathway for K^+^ to maintain basolateral K^+^ conductance [53]. McDaniel *et al*. demonstrated that Kv channel subunits (Kv1.1, Kv1.2, Kv1.3, Kv1.6, Kv7.1 and K_Ca3.1_) are expressed in IEC. Inhibition of Kv channels by 4-AP or decrease of K^+^ efflux by raising extracellular K^+^ concentration induced membrane depolarization in IEC. Maintenance of membrane depolarization would reduce the transmembrane Na^+^ driving force required for the Na^+^-dependent uptake of glucose [36]. Volker Vallon found that glucose-induced currents of small intestinal mucosa are reduced in KCNQ1^−/−^ mice compared to wild-type mice [54], indicating an important role of KCNQ1 in regulating intestinal nutrient absorption. Basolateral K^+^ channels and Na^+^/K^+^ ATPase are found to hyperpolarize membrane potential, thereby increasing the driving force for other electrogenic transport systems and maintaining the homeostasis of ionic and nutrient substances [34,55,56,57,58]. However, specific types of K^+^ channels that regulate glucose absorption and the underlying mechanisms still need further investigation.

## 5. Regulation of Glucose Absorption by Calcium Channels and Calcium-Sensing Receptors

### 5.1. Calcium Channels and Calcium-Sensing Receptors(CaSR) in Small Intestinal Epithelial Cells

Ca^2+^ plays a critical role in most physiological activities of the body, including neurotransmitter release, muscle contraction, gene regulation, cell proliferation and apoptosis [59,60,61]. Ca^2+^ homeostasis of the body is mainly maintained by intestinal Ca^2+^ absorption and renal Ca^2+^ reabsorption. Ca^2+^ entry into brush border membranes (BBM) of enterocytes is mainly through epithelial Ca^2+^ channels: transient receptor potential vanilloid (such as TRPV5 and TRPV6) and voltage-operated channels L-type voltage-gated Ca^2+^ channels (such as Ca_v_1.3) [62,63,64]. In addition, Ca^2+^ influx is also controlled through the store-operated Ca^2+^ channel (SOC) [65]. These channels are expressed in the small intestinal epithelial cells and play important roles in intestinal absorption of calcium. Besides, the role of the CaSR is non-negligible in intestinal Ca^2+^ homeostasis [66]. The CaSR is a member of the G protein‑coupled receptor (GPCR) C superfamily and is expressed in the entire digestive system [67].

### 5.2. Regulatory Mechanisms of Glucose Absorption by Calcium Channels and CaSR

It has been known that intestinal absorption of glucose is influenced by intracellular Ca^2+^ concentration; however, the underlying mechanisms are poorly understood for the Ca^2+^ regulation of glucose absorption. In the past study, Hyson and colleague observed that the kinetics of glucose uptake by SGLT1 were increased in the animals given two different types of calcium channel blockers, nisoldipine and verapamil [68]. Recently, a new model for Ca^2+^ regulation of intestinal glucose absorption has been proposed. When luminal glucose concentrations are low, intestinal glucose absorption is still mainly mediated by SGLT1 in IEC. However, when luminal glucose is higher than the ability of SGLT1 transport, the basal GLUT2 will be inserted at the apical cell membrane to participate in the absorption of glucose. These processes are regulated by [Ca^2+^]_cyt_, which also modulates GLUT2 expression in IEC [69,70]. Ca_v_1.3 has been found to play a role in the regulation of Ca^2+^ absorption through glucose stimulation [71]. Studies have pointed out that the translocation of GLUT2 from the intracellular pool is triggered by the SGLT1-dependent activation of protein Kinase C and MAP-Kinase pathways, which are associated with [Ca^2+^]_cyt_ [72]. Morgan *et al*. also demonstrated that intestinal Ca^2+^ absorption via Cav1.3 does indeed play an important role in the regulation of intestinal glucose absorption by controlling apical GLUT2 insertion [73]. Subsequently, Morgan *et al*. found that depolarization of the apical membrane by glucose absorption through SGLT1 actives the influx of luminal Ca^2+^ via Ca_v_1.3 [74]. An increase in [Ca^2+^]_cyt_ activates the calmodulin-dependent myosin light chain kinase (MLCK), further activating myosin II in the terminal web by phosphorylation of the regulatory light chain (RLC20) at Ser19. These biological processes lead to cytoskeletal rearrangement and eventually result in the translocation of GLUT2 to apical cell membranes [14,75].

The CaSR plays an important role in the regulation of [Ca^2+^]_cyt_. Activation of the CaSR on apical or basolateral membranes results in a rise in [Ca^2+^]_cyt_, which is mediated by the generation of inositol 1,4,5-trisphosphate IP_3_ via phospholipase C (PLC) and then the release of Ca^2+^ from a thapsigargin-sensitive pool [76,77]. Intracellular Ca^2+^ binds with CaM as a compound, activating a plethora of enzymes, including calcium/calmodulin-dependent protein kinase 2 (CaMKK2) [78]. David Carling and colleagues have demonstrated that pharmacological inhibition of CaMKK or downregulation of CaMKKβ using RNA interference almost completely suppresses AMPK activation [79], a key sensor of energy status within the cell that has been demonstrated to play roles in the regulation of cellular glucose uptake. Currently, regulation of glucose uptake by AMPK is controversial. Some scholars support that AMPK enhances cellular glucose uptake through the increased translocation of GLUT2 to the brush border membrane(BBM) [80,81], whereas other scholars hold that AMPK increases cellular glucose uptake via raising the expression of SGLT1 in cell membranes [82].

## 6. Regulation of Glucose Absorption by Transporters

### 6.1. Transporters in Small Intestinal Epithelial Cells

Several transporters play vital roles in maintaining the electrophysiological characteristics of small intestinal epithelial cells. Na^+^/Ca^2+^ exchangers (NCX) and sodium hydrogen exchangers (NHE) are found to express in the small intestinal epithelium [83]. NHE is a major non-nutritive Na^+^ absorptive pathway of the intestine. Nine members of the NHE family were cloned. NHE plays a key role in regulating the intracellular pH and cell volume and the referring event during such processes including cell proliferation, differentiation, apoptosis and so on [84,85,86]. Plasma membrane NHE characterized to date in animal cells utilizes the inward Na^+^ gradient created by the activity of Na^+^/K^+^-ATPase to extrude H^+^ against its electrochemical gradient in an electroneutral fashion. It is therefore not surprising that NHE3 can regulate the activity of SGLT1 and then intestinal glucose absorption [50]. On the other hand, the NCX family exists in three subtypes (NCX1-3) with different tissue distributions, which plays a role in the regulation of duodenal mucosal ion transport and HCO_3_^−^ secretion by controlling Ca^2^^+^ homeostasis [87]. The role of Na^+^/Ca^2+^ exchangers in the intestinal tract remains unclear.

### 6.2. Regulatory Mechanisms of Glucose Absorption by Transporters

It is well known that Na^+^ enters IEC via two primary pathways: SGLT1 and NHE3, which are expressed on the apical side of the small intestinal epithelium [88]. Using small interfering RNA to silence NHE3 significantly increases the SGLT1 activity of the brush border membrane in rat small intestinal cell (IEC-18) monolayers due to an increase in the number of SGLT1 [89], indicating NHE3 can regulate glucose absorption by modulating the expression of SGLT1. The regulatory mechanism of glucose absorption by NHE3 may be associated with the MAPKe-activated protein kinase 2 (MAPKAPK-2), which is activated by p38 mitogen-activated protein kinase (p38 MAPK) and finally directly phosphorylates Akt2 as well as NHE3 translocation to mediate Na^+^ absorption and Na^+^-glucose cotransport [90]. In another way, it was also found that elevated [Ca^2+^]_cyt_ can inhibit NHE3 activity to regulate Na^+^ absorption in small intestinal brush border [91]. Therefore, NHE3 regulation of intestinal glucose absorption could be carried out by different mechanisms.

NCX1 is mainly responsible for the cellular balance of Ca^2+^ and Na^+^. NCX1 can operate in either a forward mode (Ca^2+^ exit) or in a reversed mode (Ca^2+^ entry), depending on the Na^+^ and Ca^2+^ gradients and the potential across the plasma membrane [92]. The [Ca^2+^]_cyt_ and [Na^+^]_cyt_ in IEC is regulated by NCX1 expressed on both apical and basolateral sides [64,83,93]. Since [Ca^2+^]_cyt_ and [Na^+^]_cyt_ play important roles in the regulation of intestinal glucose absorption, NCX1 may be also involved in this regulatory mechanism. Unfortunately, there is no information on NCX1 regulation of intestinal glucose absorption, which might be a new direction for future research.

## 7. Conclusions

Small intestine absorption of excess glucose is associated with obesity and diabetes. However, at present, studies on the regulatory mechanisms of intestinal glucose absorption are very limited and mainly concentrate on SGLT1 [94]. Recent growing evidence suggests the involvement of cation channels, transporters and CaSR in the regulation of intestinal glucose absorption. However, further studies are needed to confirm their contributions to the regulation of glucose uptake and to elucidate the underlying mechanisms. If cation channels and transporters in IEC are confirmed to truly play important roles in modulating intestinal glucose absorption, they may become novel targets for drug discovery to treat obesity and diabetes, which are common and serious human diseases.
